# Parsonage-Turner Syndrome Following Lumbar Laminectomy and Discectomy: A Unique Case

**DOI:** 10.7759/cureus.69997

**Published:** 2024-09-23

**Authors:** Diego Martinez Castaneda, Makenzie Chambers, Catherine Fedorka

**Affiliations:** 1 Orthopaedics, Cooper Medical School of Rowan University, Camden, USA; 2 Orthopaedics, Cooper University Hospital, Camden, USA

**Keywords:** brachial neuropathy, idiopathic brachial plexopathy, lumbar discectomy, lumbar laminectomy, parsonage-turner syndrome, pts

## Abstract

We describe the case of a 32-year-old female who developed bilateral Parsonage-Turner syndrome (PTS) following laminectomy/discectomy and fusion of L4-L5. As an underdiagnosed condition, this case serves as a critical reference for physicians to recognize and include PTS in their differential diagnosis when patients present with similar symptoms following a surgery with prone positioning. This can aid physicians to make a prompt diagnosis and begin proper treatment. Finally, this case also reinforces the importance of proper positioning in the operating room.

## Introduction

Parsonage-Turner syndrome (PTS), also known as acute brachial neuropathy, idiopathic brachial plexopathy, and multiple neuritis of the shoulder girdle, is a rare inflammatory disorder of the brachial plexus. PTS typically presents with shoulder pain, motor weakness, sensory deficits, and muscular atrophy [1]. A hereditary form of the syndrome, hereditary neuralgic amyotrophy, has also been reported in the literature. Clinically, hereditary neuralgic amyotrophy presents similarly to PTS yet usually at a younger age and has a higher incidence of recurrent neuropathies [2]. The peripheral nerve involvement of PTS can vary from patient to patient but usually involves the upper trunk of the brachial plexus [1].

PTS is a clinical diagnosis, and there is currently no ancillary test that can undoubtedly diagnose the condition. Clinical history, symptoms, and physical exam findings are essential for making the correct diagnosis and can be aided by the results of magnetic resonance imaging (MRI) and electromyography (EMG) studies [3]. MRI studies of the upper extremities can demonstrate changes in the upper girdle muscles and can be used to rule out other possible shoulder pathology. EMG studies usually showcase denervation of the shoulder girdle and upper extremity muscles [4]. Currently, there is no specific management protocol for PTS, and most of the treatment focuses on symptom management, including nonsteroidal anti-inflammatory drugs (NSAIDs), steroids, and physical therapy [5,6].

Although the exact cause of PTS is complex and incompletely understood, this neurological syndrome has been reported following viral infections, immunization, and trauma [1,7]. PTS has also been associated with various surgical procedures, including hysterectomy, oral surgery, bypass surgery, and knee surgery. The etiology of PTS after a surgical procedure is felt to be due to either a traction injury to the brachial plexus or an autoimmune reaction [8]. In our literature search, we only found three reported cases of PTS following lumbar spine surgery [9-11]. Due to the scarcity of literature on this condition following lumbar spine surgery, we aim to describe a case so clinicians can be aware of this possible etiology of acute shoulder pain and weakness following surgery.

## Case presentation

A 32-year-old female with a past medical history of obesity (BMI 35.88), sciatica, spinal stenosis, tobacco use (5 pack-year), and L1 laminectomy presented to the emergency department with acute left lower extremity weakness and progressive pain. MRI of the lumbar spine demonstrated recurrence of her L4-L5 disc herniation. She was taken for an L4-L5 laminectomy/discectomy and fusion. She had no history of COVID-19 infection prior to the procedure, confirmed by COVID-19 PCR, or other viral infections. She had not had any recent vaccinations. She was positioned prone for the procedure. The surgery was unremarkable and noted to have no complications, but the patient developed right upper extremity weakness immediately after the procedure. The patient described this pain as similar to pain from a pulled muscle. The right shoulder pain shortly resolved, but then excruciating pain started in her left shoulder and arm. She had severe motor weakness in her left arm and was unable to lift it. She was prescribed oxycodone and NSAIDs which did not significantly reduce her pain. A few days later, she began to experience pain in the right shoulder once again. She had severe motor weakness in her right upper extremity and she was unable to pick up a drink of water without shaking and spilling. The patient further complained of being unable to sleep due to the excruciating pain. The left shoulder pain was located over the lateral aspect of the left shoulder and radiated down into the elbow (C6-C7 dermatome). The right shoulder pain was localized to the posterior aspect of the shoulder and radiated down toward the elbow (C7-C8 dermatome). The patient denied any paresthesia or previous surgical history on either shoulder. 

A physical exam revealed a limited neck range of motion with the absence of radicular symptoms. The patient experienced contralateral pain when laterally flexing her neck in both directions. She had reduced strength over C5, C6, and T1 (MRC 4-), but most significantly over C7 and C8 (MRC 3-). She had a full passive range of motion, but she experienced a decreased active range of motion on both shoulders. On her right shoulder, she could forward flex actively to 80 degrees, abduct to 90 degrees, and externally rotate to 40 degrees. In her left shoulder, she could forward flex actively to 120 degrees, abduct to 90 degrees, and externally rotate to 40 degrees. Reduced external rotation (MRC 4-) and abduction (MRC 4-) and internal rotation (MRC 4-) of the left and right shoulders were also noted. 

**Figure 1:**
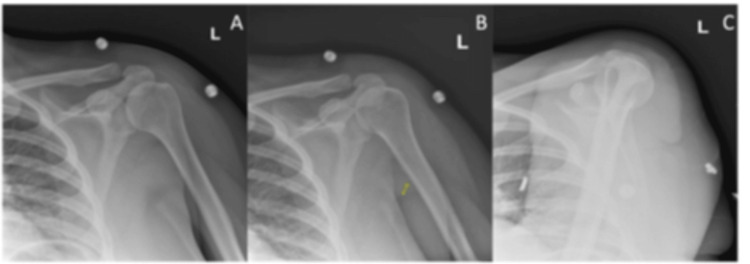
X-ray of the left shoulder showed a normal acromioclavicular joint and no acute osseous abnormalities, lesions, or arthritic changes. (A) Left shoulder external view. (B) Left shoulder internal view. (C) Left shoulder axial view.

**Figure 2:**
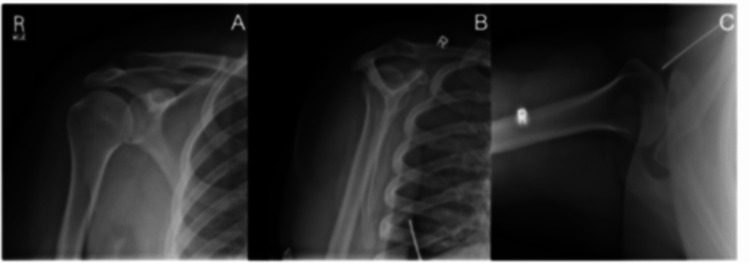
X-ray of the right shoulder showed a normal acromioclavicular joint and no acute osseous abnormalities, fractures, dislocations, or arthritic changes. (A) Right shoulder anterior-posterior view. (B) Right shoulder posterior-anterior view. (C) Right shoulder axial view.

 Left and right shoulder X-rays were normal (Figure 1 and Figure 2).

**Figure 3:**
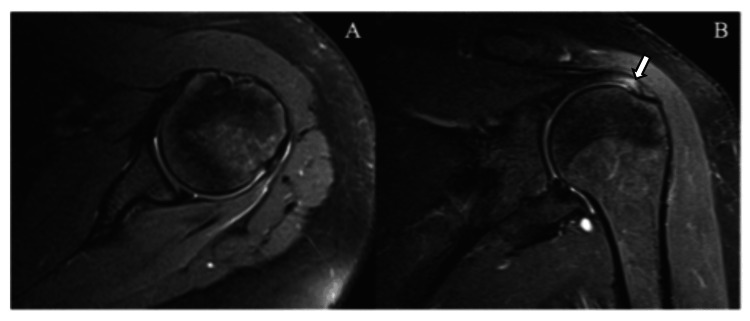
MRI of the left shoulder showed mild rotator cuff tendinosis and the absence of a tear. (A) Axial MRI. (B) Coronal MRI with an arrow pointing to tendinopathy. MRI: magnetic resonance imaging

MRI of the left shoulder performed showed mild rotator cuff tendinosis, but no tear (Figure 3).

EMG of both shoulders done a month after the procedure showed denervation potentials in the bilateral deltoid, triceps, and extensor carpi radialis longus, as well as the left infraspinatus muscle (Table 1).

**Table 1:** EMG one month post-surgery demonstrated acute denervation potentials in the bilateral deltoid, triceps, and extensor carpi radialis longus, as well as the left infraspinatus muscle. EMG: electromyography; Ins Act: insertional activity; Fibs: fibrillation potentials; PSW: peak sharp waves; Amp: amplitude; Dur: duration; Poly: polyphasic potentials; Recrt: reduced recruitment; Int Pat: interference pattern; Nml: normal; Inc: increased; 0: no fibrillation potentials; +1: persistent single trains of potentials in at least two areas

Side	Muscle	Nerve	Root	Ins Act	Fibs	Psw	Amp	Dur	Poly	Recrt	Int Pat
Right	Deltoid	Axillary	C5-C6	Nml	1+	1+	Nml	Nml	Nml	Nml	Nml
Right	Infraspinatus	SupraScap	C5-C6	Nml	1+	1+	Nml	Nml	Nml	Nml	Nml
Right	Biceps	Musculocut	C5-C6	Nml	0	0	Nml	Nml	Nml	Nml	Nml
Right	Triceps	Radial	C6-C7-C8	Nml	1+	1+	Nml	Nml	Nml	Nml	Nml
Right	ExtCarRadLong	Radial	C6-C7	Inc	0	0	Nml	Nml	Nml	Nml	Nml
Right	FlexCarRad	Median	C6-C7	Nml	0	0	Nml	Nml	Nml	Nml	Nml
Right	1stDorInt	Ulnar	C8-T1	Nml	0	0	Nml	Nml	Nml	Nml	Nml
Right	FlexPolLong	Median (Ant Int)	C7-C8	Nml	0	0	Nml	Nml	Nml	Nml	Nml
Left	Deltoid	Axillary	C5-C6	Nml	1+	1+	Nml	Nml	Nml	Nml	Nml
Left	Biceps	Musculocut	C5-C6	Nml	0	0	Nml	Nml	Nml	Nml	Nml
Left	Triceps	Radial	C6-C7-C8	Inc	0	0	Nml	Nml	Nml	Nml	Nml
Left	ExtCarRadLong	Radial	C6-C7	Nml	1+	1+	Nml	Nml	Nml	Nml	Nml
Left	FlexCarRad	Median	C6-C7	Nml	0	0	Nml	Nml	Nml	Nml	Nml
Left	1stDorInt	Ulnar	C8-T1	Nml	0	0	Nml	Nml	Nml	Nml	Nml
Left	FlexPolLong	Median (Ant Int)	C7-C8	Nml	0	0	Nml	Nml	Nml	Nml	Nml
Left	Infraspinatus	SupraScap	C5-C6	Inc	1+	1+	Nml	Nml	Nml	Nml	Nml

Clinical presentation, physical exam findings, and MRI and EMG results suggested the diagnosis of PTS and suggested the etiology was related to lumbar spine surgery. Treatment consisted of initiating physical therapy and corticosteroids to improve motor weakness and reduce inflammation. Follow-up appointments at the three-month and six-month intervals yielded a slow resolution of her weakness in both arms with physical therapy; however, her pain was still bothersome. At her one-year follow-up, she resumed work. She established care with pain management and underwent bilateral brachial plexus blocks in addition to the use of tramadol and Lyrica. She follows a home exercise program and reports a better range of motion, but her pain has persisted. Prior to the surgery, the patient had no recent history of opioid consumption, and at her final follow-up visit, she was prescribed 50 mg of tramadol. Despite the multimodal pain management, she reported waking up at night 1-2 times a week and being unable to move her right arm, with the weakness sometimes lasting all day. EMG of both shoulders was repeated at the one-year mark and demonstrated no acute denervation potentials in any muscles. There were some minimal chronic appearing positive sharp waves noted only in the right flexor carpi radialis muscles suggestive of a chronic process with some degree of reinnervation (Table 2).

**Table 2:** EMG one year post-surgery demonstrated evidence of chronic, bilateral, improving brachial plexus injury with some degree of reinnervation. EMG: electromyography; Ins Act: insertional activity; Fibs: fibrillation potentials; PSW: peak sharp waves; Amp: amplitude; Dur: duration; Poly: polyphasic potentials; Recrt: reduced recruitment; Int Pat: interference pattern; Nml: normal; Inc: increased; 0: no fibrillation potentials; +1: persistent single trains of potentials in at least two areas; Comp: complete; Mild Red: mildly reduced

Side	Muscle	Nerve	Root	Ins Act	Fibs	Psw	Amp	Dur	Poly	Recrt	Int Pat
Right	Deltoid	Axillary	C5-C6	Nml	0	0	Nml	Nml	Nml	Nml	Mild Red
Right	Biceps	Musculocut	C5-C6	Nml	0	0	Nml	Nml	Inc	Nml	Comp
Right	Triceps	Radial	C6-C7-C8	Nml	0	0	Nml	Nml	Inc	Nml	Comp
Right	ExtCarRadLong	Radial	C6-C7	Nml	0	0	Nml	Nml	Nml	Nml	Mild Red
Right	FlexCarRad	Median	C6-C7	Nml	0	1+	Nml	Nml	Inc	Nml	Comp
Right	1stDorlnt	Ulnar	C8-T1	Nml	0	0	Nml	Nml	Nml	Nml	Comp
Left	Deltoid	Axillary	C5-C6	Nml	0	0	Nml	Nml	Nml	Nml	Comp
Left	Biceps	Musculocut	C5-C6	Nml	0	0	Nml	Nml	Nml	Nml	Comp
Left	Triceps	Radial	C6-C7-C8	Nml	0	0	Nml	Nml	Nml	Nml	Mild Red
Left	ExtCarRadLong	Radial	C6-C7	Nml	0	0	Nml	Nml	Nml	Nml	Comp
Left	FlexCarRad	Median	C6-C7	Nml	0	0	Nml	Nml	Inc	Nml	Comp
Left	1stDorlnt	Ulnar	C8-T1	Nml	0	0	Nml	Nml	Nml	Nml	Comp

The patient was advised to continue her home exercise program and follow up with pain management. The patient consented to the use of the data and results pertaining to their case for publication.

## Discussion

PTS is typically a diagnosis of exclusion, and the association of this condition with surgical procedures has been extensively reported in the literature [8]. In addition, it has been reported that the onset of PTS symptoms after surgery can be quite variable, ranging from hours to several days during the postoperative period [8,12]. Although the presentation of PTS varies between cases, most patients present with a characteristic pattern of severe shoulder girdle pain preceded by motor weakness [13]. In addition, muscular atrophy and sensory deficiency are present in some instances [13]. The most commonly affected muscles are the deltoid, supraspinatus, infraspinatus, serratus anterior, biceps, and triceps [9]. Other muscles that can be affected, yet are less common, are the forearm muscles [1]. 

PTS is an uncommon condition following lumbar surgery. Some common complications following lumbar laminectomy/discectomy are dural tears, infections, epidural hematoma, intracranial hypertension during surgery, and cauda equina syndrome [4,14]. PTS has been reported in the literature following cervical spine surgery [15-17]. However, in our literature search, we only found two cases of unilateral PTS and one case of bilateral PTS reported after lumbar surgery. One case reported the onset of this neurological condition due to a tightly placed endotracheal tube during lumbar surgery [9]. Other studies reported the onset of bilateral PTS after lumbar laminectomy/fusion and unilateral PTS after lumbar discectomy, respectively [10,11]. Rubin discussed that being in a prone position for an extended period increases the risk of the superficial nature of the brachial plexus to compression or stretching injury [11]. Anderton et al. further discussed the possibility of brachial plexus neuropathy after lumbar surgery due to forceful rotation of the head, compression of the plexus between the clavicle and the first rib, as well as hyperabduction of the arms during surgical positioning, especially over 90 degrees [10]. 

In the case we describe, PTS was likely due to lumbar spine surgery. Given that it has been a year and the patient hasn't fully recovered, this could also potentially be a brachial plexus injury due to hyperabduction damage during the surgical procedure. However, the patient was carefully positioned prone on a Jackson table with her head in a neutral position and her arms abducted (60-90 degrees), and care was taken not to forcefully rotate the head or hyperabduct the arms. The elbow was flexed 90 degrees, and the arm was placed next to her head. Additionally, her symptoms did not start immediately after surgery in both arms. The symptoms on the right arm started right after surgery but the left was a delayed presentation which does not support positioning as the singular source.

## Conclusions

The physical exam findings and EMG and MRI studies, along with the presentation, are more supportive of the diagnosis of PTS. To our knowledge, this is the second reported case of bilateral PTS after lumbar laminectomy/discectomy. It is essential that physicians include PTS in their differential diagnosis when patients present with upper extremity motor weakness after lumbar surgery to ensure proper treatment. 
